# Chapter 8: Biological Knowledge Assembly and Interpretation

**DOI:** 10.1371/journal.pcbi.1002858

**Published:** 2012-12-27

**Authors:** Ju Han Kim

**Affiliations:** 1 Division of Biomedical Informatics, Seoul National University College of Medicine, Seoul, Korea; 2 Seoul National University Biomedical Informatics (SNUBI), Seoul National University College of Medicine, Seoul, Korea; 3 Systems Biomedical Informatics National Core Research Center (SBI-NCRC), Seoul National University College of Medicine, Seoul, Korea; Whitehead Institute, United States of America; University of Maryland, Baltimore County, United States of America

## Abstract

Most methods for large-scale gene expression microarray and RNA-Seq data analysis are designed to determine the lists of genes or gene products that show distinct patterns and/or significant differences. The most challenging and rate-liming step, however, is to determine what the resulting lists of genes and/or transcripts biologically mean. Biomedical ontology and pathway-based functional enrichment analysis is widely used to interpret the functional role of tightly correlated or differentially expressed genes. The groups of genes are assigned to the associated biological annotations using Gene Ontology terms or biological pathways and then tested if they are significantly enriched with the corresponding annotations. Unlike previous approaches, Gene Set Enrichment Analysis takes quite the reverse approach by using pre-defined gene sets. Differential co-expression analysis determines the degree of co-expression difference of paired gene sets across different conditions. Outcomes in DNA microarray and RNA-Seq data can be transformed into the graphical structure that represents biological semantics. A number of biomedical annotation and external repositories including clinical resources can be systematically integrated by biological semantics within the framework of concept lattice analysis. This array of methods for biological knowledge assembly and interpretation has been developed during the past decade and clearly improved our biological understanding of large-scale genomic data from the high-throughput technologies.

**What to Learn in This Chapter**

- How to find genes associated with a particular disease (or condition) from microarray or RNA-Seq data
- How to find biological pathways and/or biomedical ontology terms for the interpretation of particular gene groups associated with a particular disease
- How to characterize biological properties of a particular list of genes
- Which data resources are useful for interpreting large-scale gene expression profiles
- What are the limitations of individual gene-based analysis for determining differentially expressed genes (even with multiple hypothesis correction)
- How to identify gene groups that are differentially expressed or differentially co-expressed between normal and disease samples
- Compare in terms of semantic interpretation the functional annotation analysis methods for co-expressed genes as in clustering and for pre-defined gene sets as in GSEA
- How to organize and visualize a massive and redundant annotation list of genes or gene sets into a unified framework of biological understanding

This article is part of the “Translational Bioinformatics” collection for *PLOS Computational Biology*.

## 1. Introduction

One of the challenges in DNA microarray and RNA-Seq data analysis is to extract biological meanings from the massive amounts of transcriptome expression data. Most of the microarray and RNA-Seq data analysis methods are designed to determine the lists of genes or gene products that show distinct patterns and/or significant differences. Clustering and differential expression analysis, for example, typically generate lists of ‘significantly’ clustered and Differentially Expressed Genes (DEGs), respectively. The most challenging and rate-liming step, however, is to determine what the resulting lists of genes or gene products biologically mean.

The first analytic approach for the biological interpretation of obtained gene lists was to manually collect and put down all available descriptive information concerning each gene next to it and to try to infer the collective meaning of the textual descriptors for the group of genes under the biological systems context. The assumption here is that if a certain keyword is significantly over-represented or a meaningful pattern is found among the textual descriptors for a gene group, then the keyword or the pattern can be regarded as the semantic interpretation of the gene group.

It seems that Tavazoie *et al*. [1] was first to formally analyze the over-representation of ‘functional annotations’ for the lists of genes with semantic interpretations. By means of partitional clustering and motif discovery, given genome-wide gene-expression clusters, he analyzed significantly over-represented regulatory motifs in the upstream sequences of clustered yeast genes for uncovering new ‘regulons’ (i.e., sets of co-regulated genes) and their putative *cis*-regulatory elements. Here, the discovered motifs seem to be regarded as functional annotations to the corresponding genes. Many Functional Annotation Analysis (FAA) methods have been developed to test whether certain Gene Ontology (GO) terms [2] or biological pathways are significantly enriched within a particular list of genes. Many GO and biological pathway-based tools for gene expression analysis have been developed and proven to be useful [3]–[9].

FAA is an attempt to extract biological semantics from given lists of genes that are determined without considering any biological meaning but by a quantitative statistical analysis like clustering and DEG analysis methods. Gene Set Enrichment Analysis (GSEA) [10], [11], however, takes quite the reverse way. GSEA uses pre-defined gene sets with a priori established biological meanings like biological pathways. For each pre-defined gene set, GSEA tries to determine if it shows significant expression change. Therefore, what GSEA essentially tests is if the pre-defined ‘biological meaning’ assigned to the gene set shows significant change or not. It has been successfully demonstrated that GSEA can successfully detect subtle but set-wise coordinated expression changes that cannot be detected by individual gene tests [10].

The gene-set approach greatly improves biological interpretability by using pre-defined gene sets with established biological meanings. The same strategy can be applied for the analysis of differential co-expression analysis. Cho *et al*. proposed dCoxS algorithm that determines if a pair of gene sets' coordinated co-expression patterns shows significant changes across different conditions [12]. If a pair of gene sets (or pathways) shows a positive co-expression pattern in normal tissue but a negative co-expression pattern in cancer cells, then it can be assumed that the pair of gene sets may play an important role in the cancerous transformation. This dyadic relation can easily be extended to create a network of gene sets showing differential co-expression patterns across different conditions.

Sometimes, given the genomic scale, even the extracted list of biological meanings and significant functional annotations are too big and complex such that they need to be systematically organized. Ordering of obtained semantics using concept lattice analysis improves biological interpretation of microarray gene-expression data. BioLattice considers gene expression clusters as objects and annotations as attributes and provides a graphical ‘executive summary’ (i.e. the context of the whole experiment) of the order relations by arranging them on a concept lattice in an order based on the set inclusion theory [13].

A wide range of tools and resources in microarray and RNA-Seq data analysis have a potential impact on personalized medicine and are invaluable in biomedical research. Integrative analysis of heterogeneous biological and clinical data is essential to discover meaningful knowledge. The construction of semantic relationships of biological resources makes it possible to unify multi-layered and heterogeneously formatted data from genome to phenome. Semantic analysis integrating gene expression profiles and annotations into a unified framework enables us to interpret complex biomedical data in a comprehensive and organized fashion.

The outline for this chapter is as follows. In Section 2, a comprehensive survey of biomedical annotation resources will be given with major ontology and biological pathway-based analysis methods. Section 3 describes gene set-wise differential expression analysis methods with its semantic interpretation power. Section 4 describes differential co-expression analysis. Finally, in Section 5, application of formal concept analysis for systematic semantic interpretation of gene expression profiles will be introduced with the following summary in section 6.

## 2. Pathway and Ontology-Based Analysis

**Figure 1:**
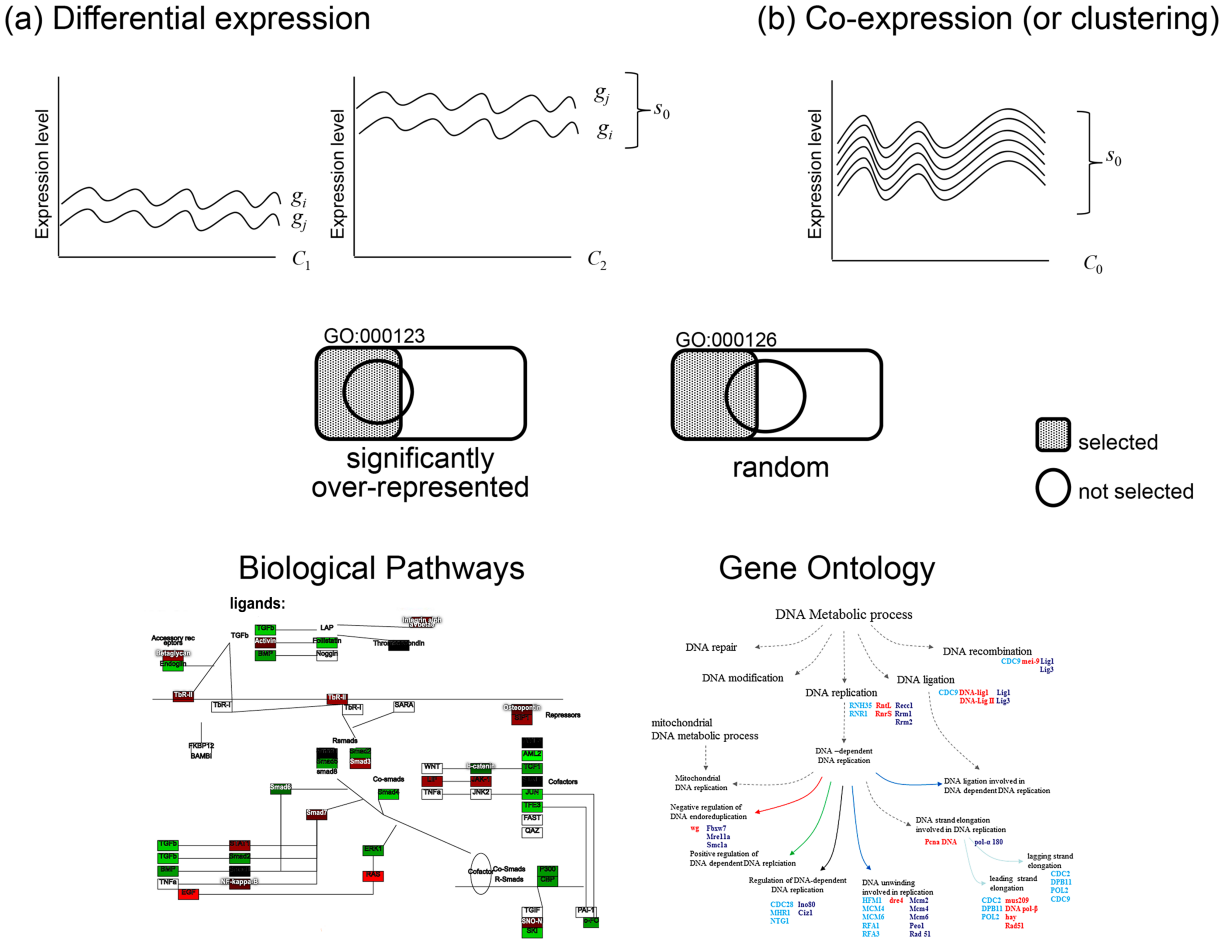
Functional annotation analysis based on biological pathways and GO terms. Annotation frequencies for a list of genes obtained by differential expression and co-expression analyses of microarray and RNA-Seq data are input to a statistical analysis of significant over-representation within the selected group. *C*: conditions, *g*: genes, *s*: gene groups.

GO and biological pathway-based analysis is one of the most powerful methods for inferring the biological meanings of observed expression changes (Figure 1). It enables us to analyze a list of interesting genes resulting from microarray and RNA-Seq experiments, without molecular biologist's help. The genes in the list may be the ones statistically significantly up or down regulated between conditions (i.e. DEGs), where the number of the genes belong to a list depends on the threshold of significance. Another method is to perform a co-expression (or clustering) analysis grouping genes with similar expression patterns across different experimental conditions.

Many genome databases provide GO annotations to their genes and gene products, which are also members of biological pathways. FAA determines which biological pathways or GO terms are significantly overrepresented in a given list of genes. GO annotation and pathway membership frequencies for a list of genes obtained by differential expression analysis (Figure 1 (a)) or co-expression analysis (or clustering) (Figure 1 (b)) are input to statistical analyses to test if they are significantly over-represented. For example, in Figure 1, the genes in the gene list (i.e. selected genes) are significantly enriched with a GO term, GO:000123, but not with GO:000126. It means that the genes are significantly associated with the biological meaning of the GO term, GO:000123.

**Figure 2:**
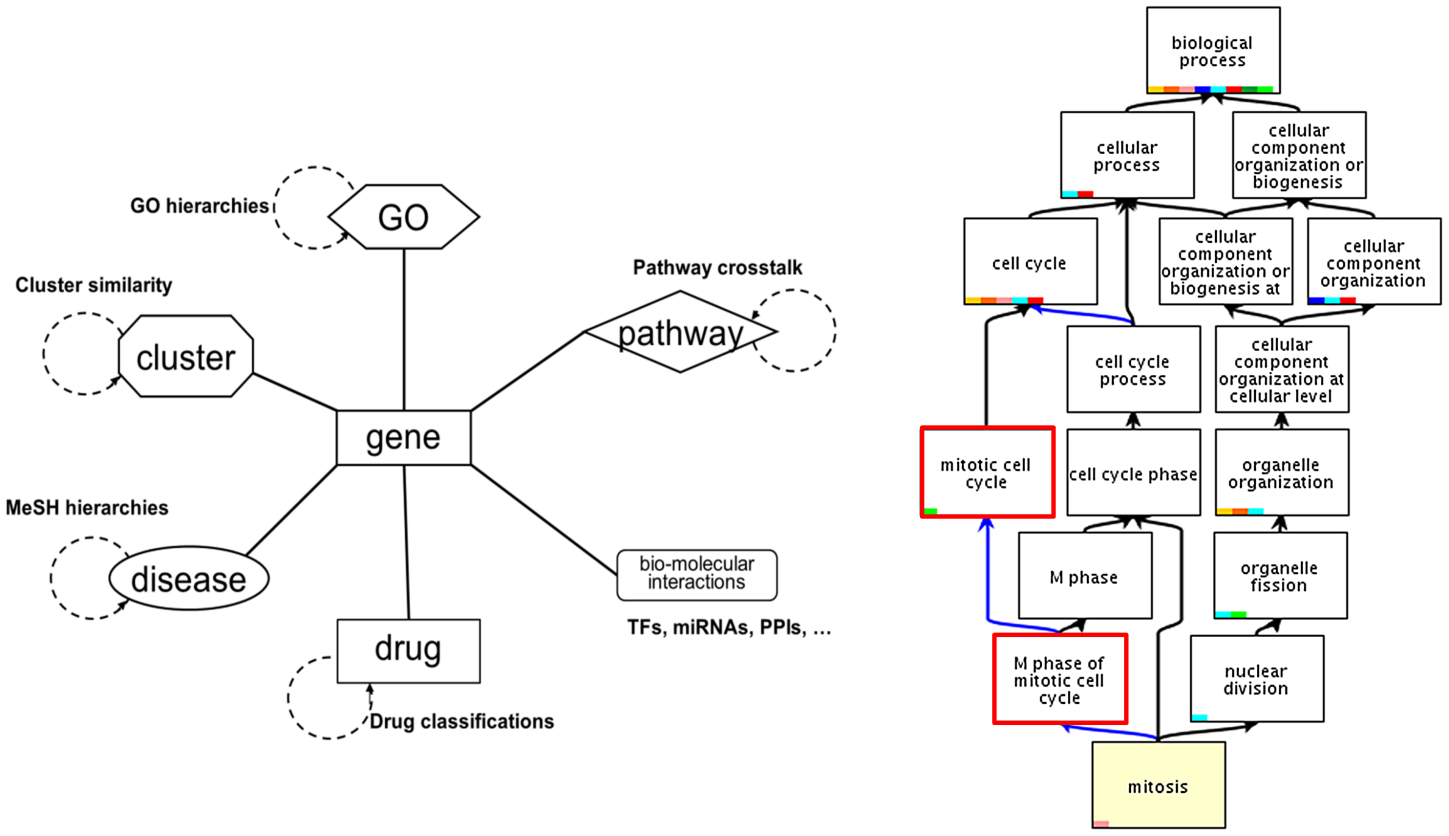
Collection of biological knowledge-based annotation resources for genes and gene clusters. The right panel shows an example of GO enrichment analysis result for a yeast cell division experiment.

In principle, any attribute of a gene can be applied for FAA including transcription factor binding sites [1], clinical phenotypes like disease associations, MeSH (Medical Subject Heading) terms, microRNA binding sites, protein family memberships, chromosomal bands, etc. as well as GO terms and biological pathways. Moreover, these features may in turn have their own ontological structures as illustrated in Figure 2. GO and MeSH have a ‘tree-ish’ graph structure, which is more formally a DAG (Directed Acyclic Graph), in which each term may be a child of one or more parents. Pathways have directed graph structures. Clusters may also be organized into a hierarchical tree or a graph structure. ArrayXPath [6], [9] provides one of the most comprehensive collections of these structured features for annotation analysis [14].

Differential expression analysis determines significantly down- or up-regulated genes (or DEGs) between two conditions, i.e. control and treatment groups to explore the effect of a drug. Student's t-test, Wilcoxon's rank sum test and ANOVA may be applied to detect DEGs. Given the huge number of genes to be tested, multiple-hypothesis-testing problem should be properly managed. Co-expression analysis puts similar expression profiles together and different ones apart, returning lists of co-expressed genes that are assumed to be tightly co-regulated. Clustering algorithms can be classified into hierarchical-tree clustering and partitional clustering. While some partitional clustering algorithms do not impose a structure to the clusters, others like Self Organizing Feature Maps (SOM) organize clusters into a grid structure. Imposing a structure based on cluster similarity may be performed after clustering.

Although DEGs are different from clusters, biological interpretation of the resulting lists of significantly up- or down-regulated DEGs (Figure 1(a)) may also be benefited by the same ontology and pathway-based annotation analysis. Clustering is classified as an unsupervised method. Results from supervised methods for a variety of classification tasks can sometimes be organized into a list based on, for example, their contributions to the task. In principle any list of genes can be carefully applied to ontology and pathway-based annotation analysis.

Metabolic pathways like KEGG and MetaCyc and signaling pathways like BioCarta are very powerful resources for the understanding of shared biological processes of a group of genes. Pathways are commonly presented as directed graphs, where nodes mainly represent molecules such as proteins and compounds, and edges represent relation types between two nodes. MetaCyc is an experimentally determined non-redundant metabolic pathway database. It is the largest collection containing over 1400 metabolic pathways [15]. It is a part of the BioCyc collection of pathways and genome databases developed by SRI International. The pathway figures of MetaCyc are not static diagrams so that it can be updated and expanded while KEGG provides static collections of pathway diagrams.

One major goal of ontology is to provide a shared understanding of a certain domain of information. GO was first created as controlled vocabularies for standardized annotation of genome databases. Genes and gene products are annotated by GO as well as free text input by curators. DAG structures are imposed to the three controlled vocabularies of GO; Molecular Function (MF), Cellular Compartment (CC), and Biological Process (BP). To each node (or GO term), a set of genes are annotated. MIPS began as a source for data on yeast biology, and now provides an integrated source for experimental, literature and computationally-predicted protein properties for a variety of complete genomes as well. MeSH has many clinical terms including disease names. Other knowledge resources like OMIM (Online Mendelian Inheritance in Man) Morbid Map can also be used to associate genes to MeSH disease names. GO and MeSH are now parts of UMLS (Unified Medical Language System) which has a semantic network structure. In principle, any biomedical ontology can be systematically applied for improving biomedical understanding of gene expression microarray and RNA-Seq data.

Once the genes of interest are successfully associated with correct functional annotations, the next step is to examine if there are any GO terms that have a larger than expected subset of listed genes in their annotation list. For example, if 20% of the genes in a gene list are annotated with a GO term ‘*apoptosis*’ while only 1% of the genes in the whole human genome fall into this functional category, then the gene list can be regarded as strongly related with the functional annotation. Most statistical tests like Chi-square, binomial and hypergeometric tests can be applied. Chi-square test cannot be used to test data of small sample size. Hypergeometric test is widely used for functional enrichment analysis of gene lists, but it is computationally more intensive.

Suppose we have a total of *N* genes with *n* genes belonging to a group of interest (cluster or DEGs). Among them *M* genes are annotated to a specific GO term and *k* genes belong to the interest group and are annotated to the specific GO term. The probability of having at most *k* genes can be calculated by hypergeometric distribution according to the following:
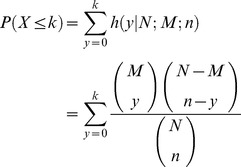



Hypergeometric distribution is a discrete probability distribution describing the number of successes by a serial sampling from a finite population. It is equivalent to a one-tailed Fisher's exact test. One should consider the choice of universe (or background), that makes substantial impact on the result. All genes having at least one GO annotation, all genes ever known in genome databases, all genes on the microarray, or all transcripts of RNA-Seq data that pass non-specific filters can be candidate universe. One more problem comes from the hierarchical tree (or graphical) structure of GO categories (or pathways) while the hypergeometric test assumes independence of categories. A parent term can simply be rated as significant because of the influence from its significant children. Moreover, more general statements require stronger evidence that is required to prove more specific statements. Conditional hypergeometric testing methods [16], [17] exclude GO terms if there is no evidence beyond that provided by its significant children. Because many tests are performed, p-values must be interpreted with caution.

Pathway and ontology-based analysis consist of database mapping, statistical testing, and presentation steps [18]. Mapping gene lists to GO terms or pathways requires resolving gene name ambiguities and inconsistencies (not discussed here) using a wide range of genomic resources and techniques. Visual and textual presentation helps users to understand biological semantics and contexts. A number of analysis tools with these steps have been introduced: ArrayXPath, Pathway Miner, EASE in pathway analysis, GOFish, GOTree Machine, FatiGO, GOAL, GOMIner, FuncAssociate in ontology analysis and GeneMerge, MAPPFinder, DAVID, GFINDer, OntoTools in both analyses [14].

## 3. Gene Set-Wise Differential Expression Analysis

Researcher's primary interest with DNA microarray and RNA-Seq data is to identify differentially expressed genes (DEGs). To this aim, a number of statistical methods have been introduced, evaluating statistical significance of individual genes between two conditions. Gene set-wise differential expression analysis method, however, evaluates coordinated differential expression of gene groups, the meaning of which are previously defined as those of biological pathways. The first developed in this category is the Gene Set Enrichment Analysis (GSEA) that evaluates for each a priori defined gene set the significant association with phenotypic classes in DNA microarray experiments [10].

**Figure 3:**
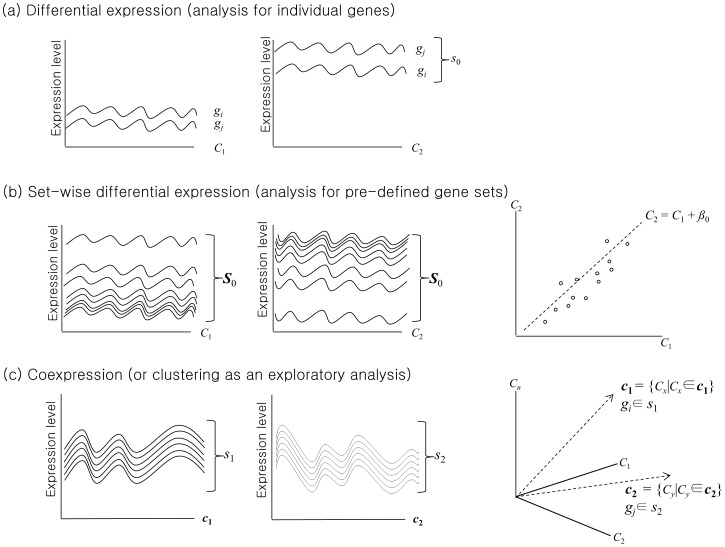
Differential expression analysis for individual genes and predefined gene sets. *C*: conditions, ***c***: condition sets, *g*: genes, *s*: gene groups, ***S***: predefined gene sets. (Modified from [30]).

While FAA tries to determine over-represented GO terms or biological pathways after determining significant co-expression clusters or DEG lists (Figure 3(a) and (c)), GSEA takes the ‘reverse-annotation’ or ‘gene set-wise’ approach (Figure 3(b)). This gene set-wise differential expression analysis method successfully identified modest but coordinated changes in gene expression that might have been missed by conventional ‘individual gene-wise’ differential expression analysis. Moreover, gene set-wise approach provides straightforward biological interpretation because the gene sets are defined by biological knowledge. GSEA's success clearly demonstrates that many tiny expression changes can collectively create a big change that is statistically significant. Another advantage is that utilizing pre-defined and well-established gene sets rather than finding or creating novel lists of genes markedly improves semantic interpretability and computational feasibility. It is believed that functionally related genes often show a coordinated expression pattern to accomplish their functional role.

GSEA first creates a ranked list of genes according to their differential expression between experimental conditions and then determines, for each a priori defined gene set, whether members of a gene set tend to occur toward the top (or bottom) of the ranked list, in which case the gene set is correlated with the phenotypic class distinction. With the interesting gene set, *S*, Enrichment Score (ES) is calculated by evaluating the fractions of genes in *S* (“hits”) weighted by their correlation and the fractions of genes not in *S* (“misses”) present up to a given position *i* in the ranked gene list, *L*, where *N* genes are ordered according to the correlation, *r*(*g_j_*) = *r_j_* of their expression profiles with interest gene set:
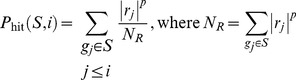


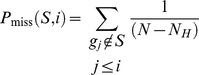
where *N_H_* indicates the number of genes in *S* and is an exponent to control the weight of the step. The ES is the maximum deviation from zero of *P*_hit_ – *P*_miss_. It corresponds to a weighted Kolmogorov-Smirnov-like statistic.

GSEA assesses the significance by permuting the class labels. Concerning the definition of the null hypothesis, methods can be classified into competitive and self-contained tests [19]. A competitive test compares differential expression of the gene set to a standard defined by the complement of that gene set. A self-contained test, in contrast, compares the gene set to a fixed standard that does not depend on the measurements of genes outside the gene set. The competitive test is more popular than the self-contained test.

Typical gene sets are regulatory-motif, function-related, and disease-related sets. MSigDB (Molecular Signatures Database) is one of leading gene set databases (http://www.broadinstitute.org/gsea/msigdb) containing a total of 6769 gene sets which are classified into five different collections (positional, curated, motif, computational and GO gene sets). Several interesting extensions were proposed in terms of sample level applications. For example, researchers developed genomic signatures to identify the activation status of oncogenic pathways and predict the sensitivity to individual chemotherapeutic drugs [20], . Significance Analysis of Function and Expression (SAFE) [22] extends GSEA to cover multiclass, continuous and survival phenotypes. It also provides more options for the test statistic, including Wilcoxon rank sum, Kolmogorov-Smirnov and Hypergeometric statistic.

## 4. Differential Co-Expression Analysis

**Figure 4:**
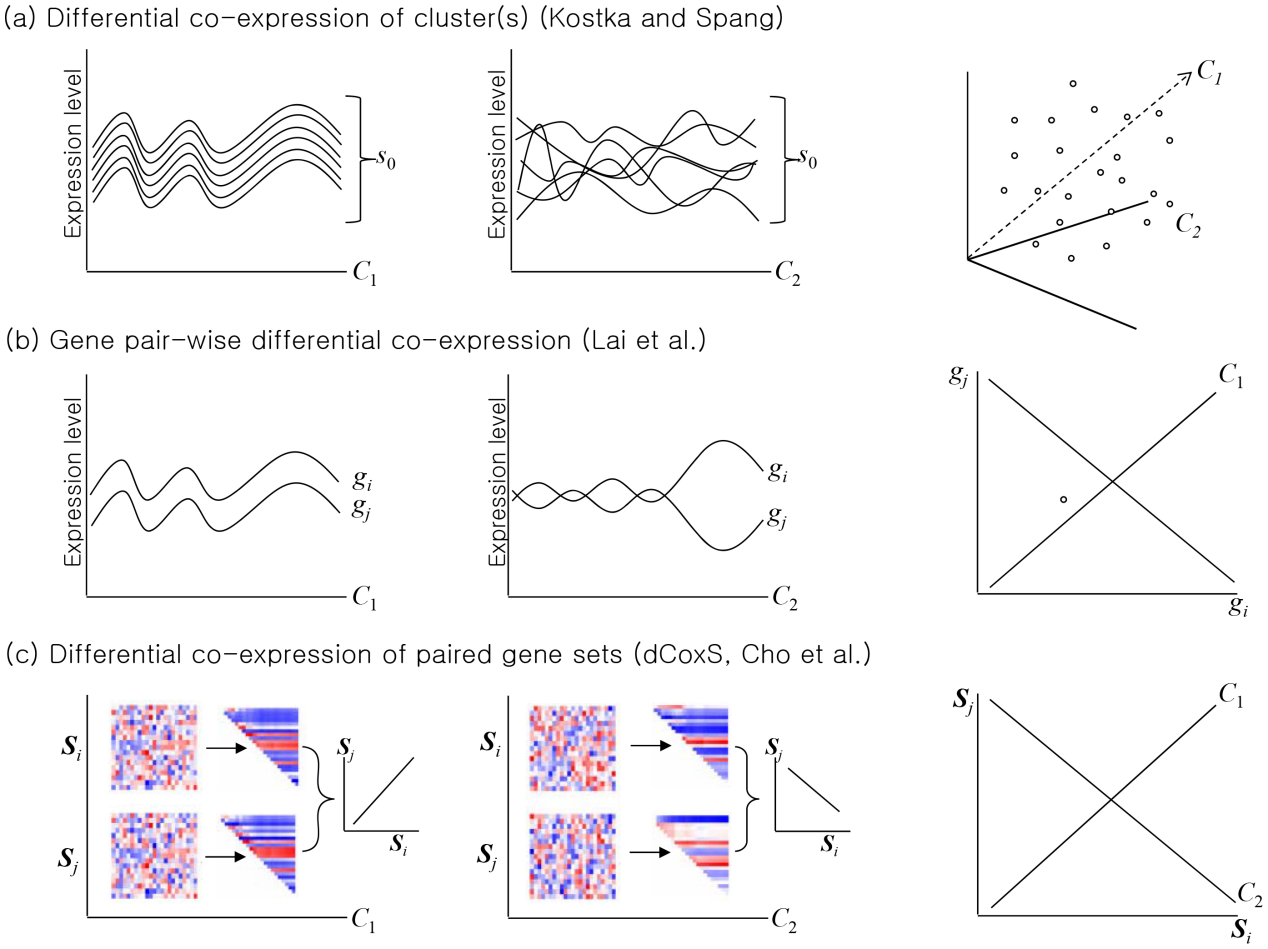
Differential co-expression anslyses. Differential co-expression (a) of clusters can be detected by a method proposed by Kostka and Spang [26], (b) of gene pairs can be detected by a method proposed by Lai *et al*. [24], and (c) of paired gene sets by a method proposed by Cho *et al*. [12]. *C*: conditions, *g*: genes, *s*: gene clusters, *S*: a priori defined gene sets. (Modified from [30]).

Co-expression analysis determines the degree of co-expression of a group (or cluster) of genes under a certain condition. Unlike co-expression analysis, differential co-expression analysis determines the degree of co-expression difference of a gene pair or a gene cluster across different conditions, which may relate to key biological processes provoked by changes in environmental conditions [12], [23]–[25]. Differential co-expression analysis methods can be categorized into three major types (Figure 4): (a) differential co-expression of gene cluster(s) [26], (b) gene pair-wise differential co-expression [24] and (c) differential co-expression of paired gene sets [12].

To identify differentially co-expressed gene cluster(s) between two conditions, (*C*_1_ and *C*_2_ in Figure 4 (a)), a method determines whether a cluster shows significant conditional difference in the degree of co-expression. An additive model-based scoring can be used based on the mean squared residual [26]. Let conditions and genes be denoted by *J* and *I*, respectively. The mean squared residual of model is a measurement of co-expression of genes:
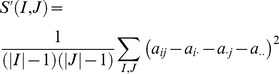
where an entry *a_ij_* is the expression level of gene *i* in condition *j*, *a_i_*. is the mean expression level of gene *i* in conditions, *a._j_* is the mean expression level of genes in condition *j*, *a*..is the mean expression levels of genes in conditions. A group of gene with a low score *S*′ means high correlation of genes. Given two groups *J*_1_ and *J*_2_, e.g. disease and control, the method minimizes the score, *S* (*I*) of a set of genes, *I*:
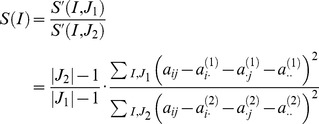



A greedy downhill approach finds local minima of the score. Another approach uses t-statistic for each cluster to evaluate the difference of the degree of co-expression between conditions, after creating gene expression clusters [27]. These methods can be viewed as an attempt to find gene clusters that are tightly co-regulated (i.e. highly co-expressed) in one condition (i.e. normal) but not in another (i.e. cancer).

To identify differentially co-expressed gene pairs in Figure 4(b), *F*-statistic can be calculated as expected conditional *F*-statistic (ECF), a modified *F*-statistic, for all pair of genes between two conditions [24]. A meta-analytic approach can also detect gene pairs with significant differential co-expression between normal and cancer samples [25]. These methodologies can be regarded as an attempt to discover gene pairs that are, in principle, positively correlated in one condition (i.e. normal) and negatively correlated in another (i.e. cancer). Identification of differentially co-expressed gene clusters or gene pairs usually do not use a priori defined gene sets or pairs but try to find the best ones among all possible combinations without considering prior knowledge. Thus the biological interpretation of the clusters or pairs may also be improved by ontology and pathway-based annotation analysis.

The idea of finding gene clusters that show positive correlation in one condition and negative correlation in another condition sounds very interesting. However, it seems that there is very little chance for such a cluster to exist. Similarly, one can hardly find such a set among a priori defined gene sets (i.e. biological pathways). It is even difficult to expect a biological pathway whose members are all highly positively (or negatively) co-expressed in a condition because a biological pathway is a complex functional system with interacting positive and negative feedback loops. Thus, members of a biological pathway may not be contained in a single co-expression cluster, especially when the cluster is not very big, but be split into different clusters.

**Figure 5:**
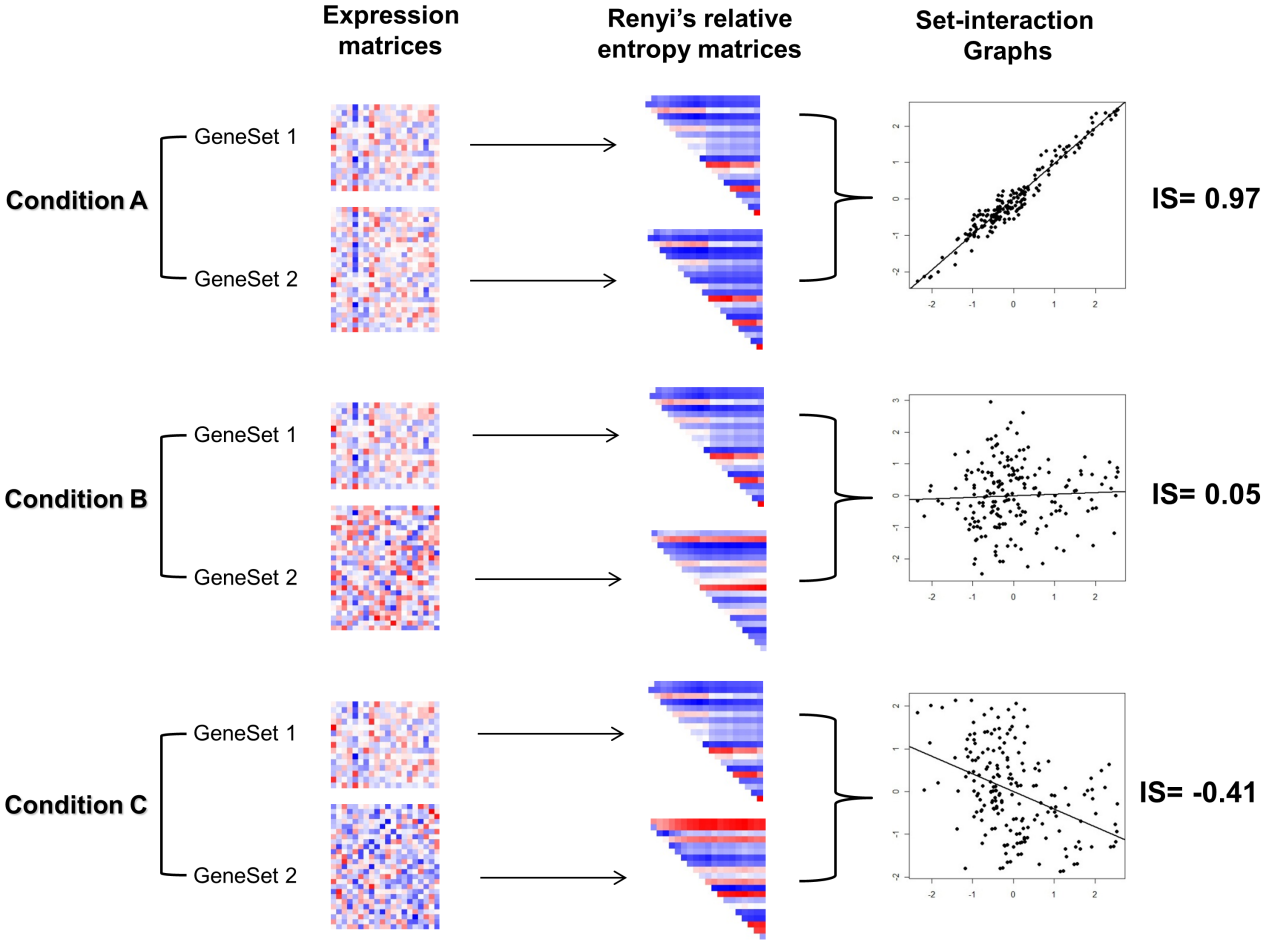
The dCoxS algorithm. Expression matrices of two gene sets (upper panel) are transformed into Renyi relative entropy matrices by all sample pair-wise comparisons (middle panel). For each condition, Interaction Score (*IS*), a kind of correlation coefficients, between a pair of entropy matrices is obtained. Upper diagonal heat maps in the middle panel are transformed into scatter plots in the lower panel where *IS*s are depicted as fitted lines. (Modified from [12]).

The dCoxS (differential co-expression of gene sets) algorithm identifies (a priori defined or semantically enriched) gene set pairs differentially co-expressed across different conditions (Figure 4 (c) and Figure 5) [12]. Biological pathways can be used as pre-defined gene sets and the differential co-expression of the biological pathway pairs between conditions is analyzed. To measure the expression similarity between paired gene-sets under the same condition, dCoxS defines the interaction score (*IS*) as the correlation coefficient between the sample-wise entropies. Even when the numbers of the genes in different pathways are different, *IS* can always be obtained because it uses only sample-wise distances regardless of whether the two pathways have the same number of genes or not.
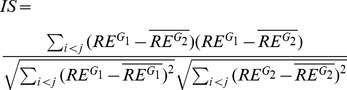
where *RE^Si^* and *RE^Sj^* are the matrices of the Renyi relative entropy of gene sets, *S_i_* and *S_j_*. When estimating the relative entropy, multivariate kernel density estimation was used to model gene-gene correlation structure.

For example, when we compute the *IS* of a pair of pathway expression matrices with dimensions 20 (genes) by 25 and by 15 (samples) for a condition, we calculate 190 ( = (20*19)/2) sample pair-wise entropy distances for each pathway expression matrix. The *IS* is obtained by calculating the correlation coefficient between the two entropy vectors. Finally, the statistical significance of the difference of the Fisher's Z-transformed *IS*s between two conditions is tested for each pathway pair.
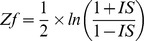



The *p*-value of the difference in the *Zf* values is calculated using the standard normal distribution in equation.
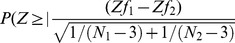



*Zf_1_* and *Zf_2_* are the Fisher's Z-transformed values of the IS under two different conditions and *N_1_* and *N_2_* are the numbers of upper-diagonal elements, which is calculated by *n*(*n*−1)/2 (*n* = number of samples) for each condition.

For the purpose of comparison, all gene pair-wise *Zf* values are calculated for each condition and the conditional difference of the Fisher's Z-transformed correlation coefficients is tested for each gene pair as follows,
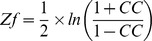


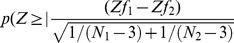
where *CC* indicates the correlation coefficient of a gene pair, *Zf_i_* Fisher's Z-transformed correlation coefficient and *N_i_* the number of samples in conditions *i*. The p value for differential co-expression is obtained according to the difference between the *Z* values from the normal distribution. For each gene pair, three p values are obtained, one from each condition and another from the difference between the conditions. Bonferroni correction is applied.

## 5. Biological Interpretation and Biological Semantics

Biological interpretation of genomic data requires a variety of semantic knowledge. Biomedical semantics provides rich descriptions for biomedical domain knowledge. Biomedical semantics is a valuable resource not only for biological interpretation but also for multi-layered heterogeneous data integration and genotype-phenotype association. Symbolic inference algorithms may add further values.

Although GO and pathway-based analysis of co-expressed gene groups is one of the most powerful approaches for interpreting microarray experiments, they have limitations. The result, for example, is typically a long unordered list of annotations for tens or hundreds of gene clusters. Most of the analysis tools evaluate only one cluster at a time in a sequential manner without considering the informative association network of clusters and annotations. It is very time-consuming to read the massive annotation lists for a large number of clusters. It is unthinkably hard to manually assemble the ‘puzzle pieces’ (i.e., the cluster-annotation sets) into an ‘executive summary’ (i.e., the context of the whole experiment). Many annotations are redundant such that many clusters share the same annotations in a very complex manner. Ideally, the assembly should involve eliminating redundant attributes and organizing the pieces in a well-defined order for better biological understanding and insight into the underlying ‘context’ of the experiment under investigation.

BioLattice is a mathematical framework based on concept lattice analysis to organize traditional clusters and associated annotations into a lattice of concepts for better biological interpretation of microarray gene-expression data [13]. BioLattice considers gene expression clusters as objects and annotations as attributes and provides a graphical summary of the order relations by arranging them on a concept lattice in an order based on set inclusion relation. Complex relations among clusters and annotations are clarified, ordered and visualized. Redundancy of annotation is completely removed. It also has an advantage that heterogeneous biological knowledge resources (such as transcription factor binding, chromosomal co-location and protein–protein interaction networks) can be added to better explore the underlying structures. The representation of relationship between clusters can give more insight to interpret functions of interesting genes.

**Figure 6:**
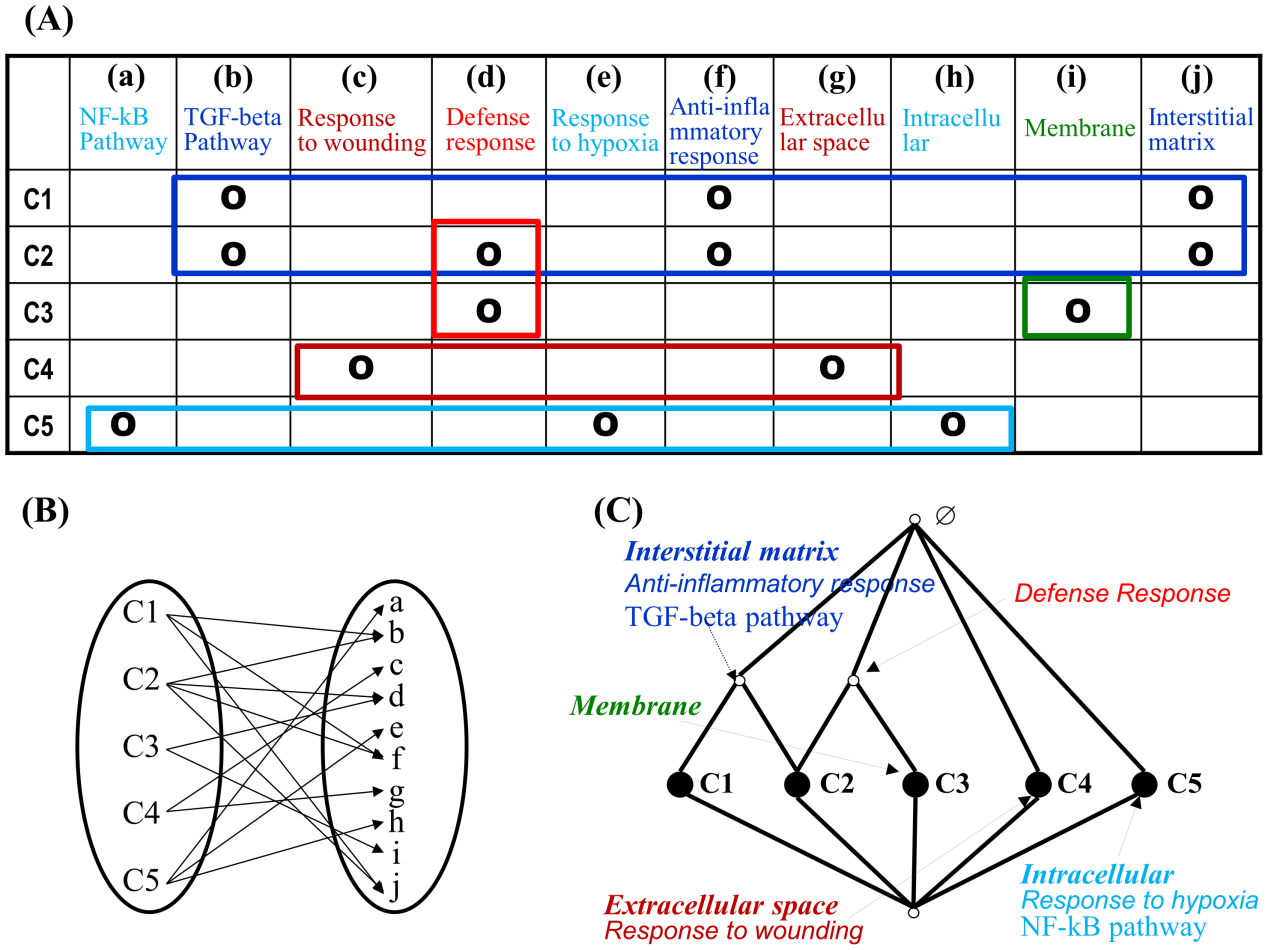
Concept lattice. The binary relation set *R* = { (*C1,b*), (*C1,f*), (*C1,j*), (*C2,b*), (*C2,d*),…, (*C5,e*), (*C5,h*)} can be represented as (a) a relation matrix, (b) a directed bipartite graph, and (c) a concept lattice. Colored rectangles in the relation matrix represent concepts. The same color represents the same concept in (a) and (c). (Modified from [13]).

Figure 6 demonstrates a context (or a gene expression dataset) with clusters and annotations. Note that the relation matrix between objects (i.e., rows or clusters) and attributes (i.e., columns or annotations) can be represented by a bipartite graph (Figure 6(b)) or a concept lattice (Figure 6(c)). A concept lattice organizes all clusters and annotations of a relation matrix into a single unified structure with no ‘redundancy’ and no loss of information. It is worth noting that the cluster labels, *C1* to *C5*, and the annotation labels appear once and only once in the lattice diagram (Figure 6(c)). Now one can interpret the whole experimental context (Figure 6(a)) by reading the ordered concepts with clusters and annotations.

**Figure 7:**
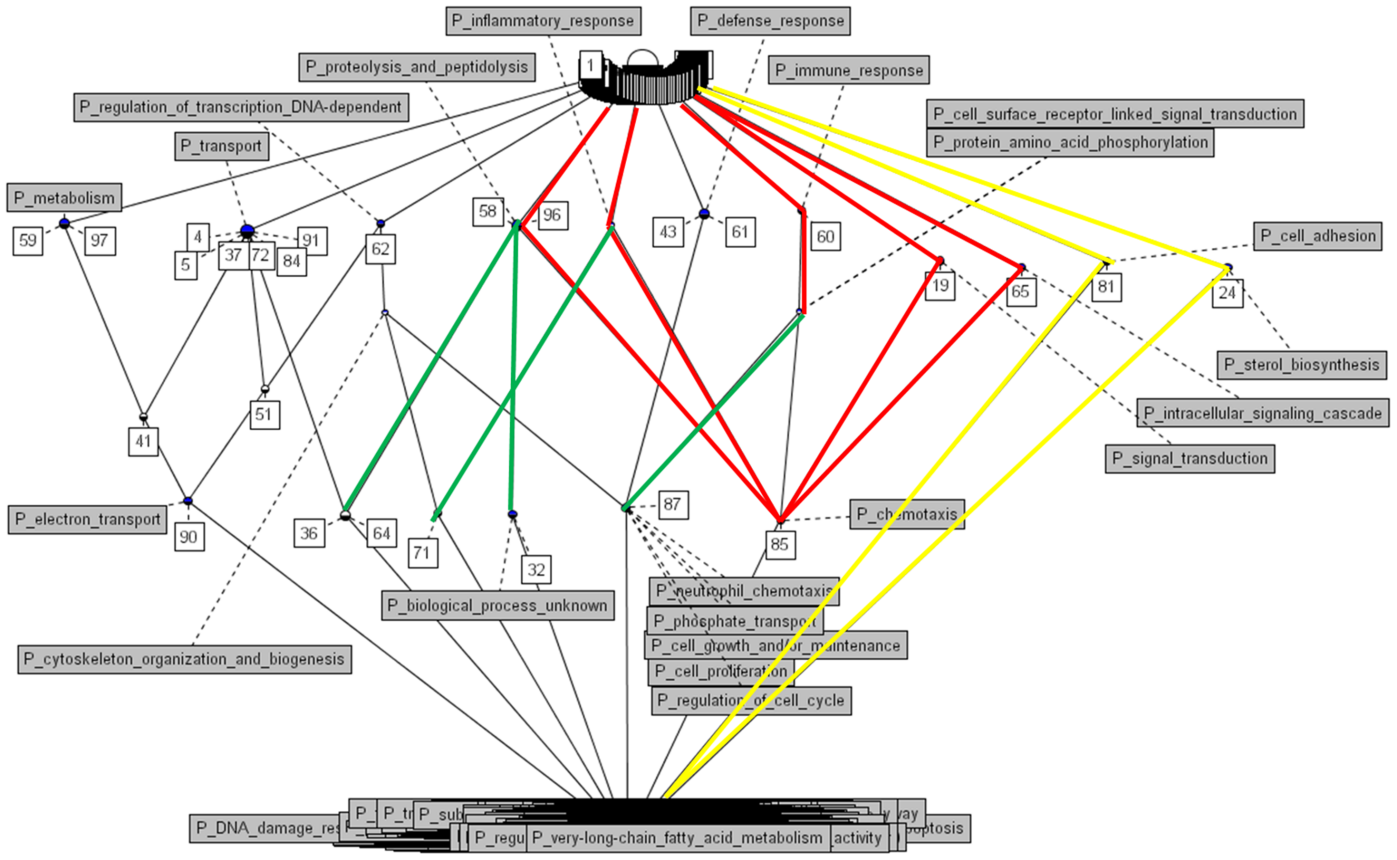
BioLattice of mouse renal inflammation induced by glomerular basement membrane (GBM) antibody.

Structural analyses methods like prominent sub-lattice analysis and core-periphery structure analysis may help further understanding [13]. Figure 7 shows a BioLattice for a mouse anti-GBM glomerulonephritis model [28]. Genes showing significant time-dose effect were clustered into 100 clusters and annotated with GO terms. The whole complex clusters and annotations are organized into a single unified lattice graph, providing an ‘executive summary.’ The Ganter algorithm [29] can be used to construct BioLattice. A web-based tool using Perl, JavaScript and Scalable Vector Graphics are available at http://www.snubi.org/software/biolattice/. Prominent sub-lattice analysis reveals a meaningful sub-structure converging into cluster 85, which has the GO term ‘*chemotaxis*’ and inherits ‘*proteolysis and peptidolysis*’ (clusters 58 and 96), ‘*inflammatory response*’, ‘*immune response*’, ‘*protein amino acid phosphorylation*’, and ‘*cell surface receptor linked signal transduction*’ (cluster 60), ‘*signal transduction*’ (cluster 19), ‘*intracellular signaling cascade*’ (cluster 65). It is clearly visualized that cellular immune response system activation is the core pathological process in the IgA nephropathy model of kidney and clusters 19, 58, 60, 65, 5 and 96 are within those concepts.

Context in concept lattice analysis is a triplet (*G, M, I*) consisting of two sets *G* and *M* and a relation *I* between *G* and *M*. The elements of *G* and *M* are called objects and attributes, respectively. We denote *gIm* or (*g*, *m*) ∈ *I* to show that object *g* has attribute *m*. For a set *A* ⊆ *G* of objects, we define *A*′: = { *m* ∈ *M* | *gIm* for all *g* ∈ *A* } (i.e., the set of attributes common to the objects in *A*). Correspondingly, for a set *B*⊆*M* of attributes, we define *B*′: = { *g* ∈ *G* | *gIm* for all *m* ∈ *B* } (i.e., the set of objects that have all attributes in *B*).

Concept lattice analysis models concepts as units of thought, consisting of two parts. A concept of the context (*G, M, I*) is a pair (*A*, *B*) with *A*⊆*G*, *B*⊆*M*, *A*′ = *B* and *B*′ = *A*. We call *A* and *B* the extent and the intent, respectively, of concept (*A*, *B*). The extent consists of all objects belonging to the concept while the intent contains all attributes shared by the objects. The set of all concepts of the context (*G, M, I*) is denoted by *C*(*G, M, I*). A concept lattice is drawn by ordering (*A*, *B*), which are defined as concepts of the context (*G, M, I*). The set of all concepts of a context together with the partial order (*A1*, *B1*)≤(*A2*, *B2*): ⇔ *A1* ⊆ *A2* (which is equivalent to *B1* ⊇ *B2*) is called a concept lattice.

We regard *A* as defining gene expression clusters that share common knowledge attributes and *B* as defining the knowledge terms that are annotated to the clusters. The concepts are arranged in a hierarchical order so that the order of *C1*≤*C2* ⇔ *A1* ⊆ *A2* ⇔ *B1* ⊇ *B2* is defined at *C1* = (*A1*, *B1*), *C2* = (*A2*, *B2*). The top element of a lattice is a unit concept, representing a concept that contains all objects. The bottom element is a zero concept having no object.

## 6. Summary

This chapter has shown major computational approaches to facilitate biological interpretation of high-throughput microarray and RNA-Seq experiments. The enrichment analysis with ontologies, biological pathways or external resources is widely used to interpret the functional role of correlated genes or differentially expressed genes. In analysis steps, the groups of genes are assigned to the associated biological annotation terms using GO terms or biological pathways. Then it is necessary to examine whether gene members are statistically enriched in each of the annotation terms or pathway by comparing background set by measuring statistical test such as Chi-square, Fisher's exact, binomial and hypergeometric test. Unlike previous approaches identifying a set of significant genes, Gene Set Enrichment Analysis uses pre-defined sets to search for groups of functionally related genes with coordinated expression across a list of genes ranked by differentially expression. Differential co-expression analysis determines the degree of co-expression difference of a gene set pair across different conditions. The dCoxS algorithm identifies differentially co-expressed gene set under different conditions. Outcomes in microarray and RNA-Seq data can be transformed into the graphical structure that represents biological semantics. A number of biomedical annotation and external repositories including clinical resources can be integrated by biological semantics analysis tools such as BioLattice.

## 7. Exercises

1. Select significantly DEGs from the train dataset of AML (Acute Myelocytic Leukemia) and ALL (acute lymphoblastic leukemia) expression data (http://www.broadinstitute.org/cgi-bin/cancer/publications/pub_paper.cgi?mode=view&paper_id=43) and find enriched GO terms from an ontology analysis tool. Dataset and analysis functions are also included in R statistical package, golubEsets in Bioconductor.
2. List significantly enriched pathways using a pathway analysis tool with the dataset in Exercise 1.
3. Find KEGG pathways significantly associated with leukemia subtype in the 2-sample comparison of AML and ALL by GSEA through the Kolmogorov-Smirnoff test. Analysis and data set are provided by SAFE R (http://bioconductor.org/packages/2.0/bioc/html/safe.html).
4. Identify the differentially co-expressed gene set pairs using dCoxS with simulated data in (http://www.snubi.org/publication/dCoxS). Compute interaction score between matrix M and M1 using ias function. And, compute interaction score between M and M2. Finally, using compcorr function, estimate significance of difference of ias. Note that in compcorr function, n1 and n2 is the number of all possible sample pairs.
5. Report semantic relationships of pathways and GO terms using BioLattice (http://www.snubi.org/software/biolattice/). Use the result of *k*-means clustering (*k* = 10) with DEG in Exercise 1. Select Category as ‘biological process,’ p-value<0.05.

Answers to the Exercises can be found in Text S1.

**Further Reading**

- Draghici S (2003) Data analysis tools for DNA microarrays. Chapman and Hall/CRC Press.
- Curtis RK, Oresic M, Vidal-Puig A (2005) Pathways to the analysis of microarray data. Trends Biotechnol 23(8): 429–435.
- Gentleman R, Carey V, Huber W, Irizarry R, Dudoit S (2005) Bioinformatics and computational biology solutions using R and Bioconductor. Springer.
- Deshmukh SR, Purohit SG (2007) Microarray data: statistical analysis using R. Oxford: Alpha Science International Ltd.
- Guerra R, Goldstein DR (2008) Meta-analysis and combining information in genetics and genomics. Chapman and Hall.
- Werner T (2008) Bioinformatics applications for pathway analysis of microarray data. Current Opinion in Biotechnology 19 (1): 50–54.
- Emmert-Streib F, Dehmer M (2010) Medical biostatistics for complex diseases. Wiley.
- Kann MG (2010) Advances in translational bioinformatics: computational approaches for the hunting of disease genes. Brief Bioinform 11(1): 96–110.

**Glossary**

**Bioconductor**: a free, open source and open development software project for the analysis and comprehension of genomic data generated by wet lab experiments in molecular biology written in R Statistical Package.

**Clustering**: algorithm that puts similar things together and different things apart.

**Gene expression profiling**: the measurement of the activity (or expression) of thousands of genes at once to create a global picture of cellular function using DNA microarray technology.

**Gene set**: a meaningful grouping of genes like biological pathways, genes sharing certain regulatory-motifs, genes sharing certain functional annotations, and certain disease-related gene sets.

**Gene Set Enrichment Analysis**: an algorithm to determine whether an a priori defined set of genes shows statistically significant coordinated differential expression between conditions.

**Gene Ontology**: a set of controlled vocabularies in molecular function, biological process and cellular component for the standardized annotations of genes and gene products across all species.

**Hypergeometric distribution**: a discrete probability distribution that describes the number of successes in a sequence of *n* draws from a finite population without replacement, just as the binomial distribution describes the number of successes for draws with replacement.

**Kolmogorov–Smirnov test (K–S test)**: a nonparametric test for the equality of continuous, one-dimensional probability distributions that can be used to compare a sample with a reference probability distribution (one-sample K–S test), or to compare two samples (two-sample K–S test).

## Supporting Information

Text S1 Answers to Exercises
(DOCX)
