# Supplementary material for: Chapter 8: Biological Knowledge Assembly and Interpretation
Source: PLoS Comput Biol. 2012 Dec 27;8(12):e1002858. doi: 10.1371/journal.pcbi.1002858 (PMC3531281; doi:10.1371/journal.pcbi.1002858)
Supplement: Text S1 — Answers to Exercises (DOCX) [file pcbi.1002858.s001.docx]

**Answers to the Exercises**

1. Select significantly DEGs from the train dataset of AML (Acute Myelocytic Leukemia) and ALL (acute lymphoblastic leukemia) expression data (http://www.broadinstitute.org/cgi-bin/cancer/publications/pub_paper.cgi?mode=view&paper_id=43) and find enriched GO terms from an ontology analysis tool. Dataset and analysis functions are also included in R statistical package, golubEsets in Bioconductor.

2. List significantly enriched pathways using a pathway analysis tool with the dataset in Exercise 1

3. Find KEGG pathways significantly associated with leukemia subtype in the 2-sample comparison of AML and ALL by GSEA through the Kolmogorov-Smirnoff test. Analysis and data set are provided by SAFE R (<http://bioconductor.org/packages/2.0/bioc/html/safe.html>).

4. Identify the differentially co-expressed gene set pairs using dCoxS with simulated data in (<http://www.snubi.org/publication/dCoxS>). Compute interaction score between matrix M and M1 using ias fucntion. And, compute interaction score between M and M2. Finally, using compcorr function, estimate significance of difference of ias. Note that in compcorr function, n1 and n2 is the number of all possible sample pairs.

5. Report semantic relationships of pathways and GO terms using BioLattice (<http://www.snubi.org/software/biolattice/>). Use the result of *k*-means clustering (*k*=10) with DEG in Exercise 1. Select Category as ‘biological process’, p-value < 0.05.

**Answer 1:**

<DEG Gene List >

D87078_at (PUM2), D88270_at (VPREB1), J05243_at (SPTAN1), L41870_at (RB1), L47738_at (CYFIP2), M11147_at (FTL), M11722_at (DNTT), M16038_at (LYN), M21551_rna1_at (NMB), M23197_at (CD33), M27891_at (CST3), M29696_at (IL7R), M37435_at (CSF1), M55150_at (FAH), M60527_at (DCK), M62762_at (ATP6V0C), M89957_at (CD79B), M91432_at (ACADM), M94633_at (RAG2), S50223_at (ZNF22), U05259_rna1_at (CD79A), U20998_at (SRP9), U32944_at (DYNLL1), U46499_at (MGST1), U49844_at (ATR), U50136_rna1_at (LTC4S), U50928_at (PKD2), U62136_at (UBE2V2), U73737_at (MSH6), U82759_at (HOXA9), X04085_rna1_at (CAT), X15949_at (IRF2), X17042_at (SRGN), X59350_at (CD22), X61587_at (RHOG), X62535_at (DGKA), X62654_rna1_at (CD63), X63469_at (GTF2E2), X74262_at (RBBP4), X74801_at (CCT3), X82240_rna1_at (TCL1A), X95735_at (ZYX), Y12670_at (LEPROT), Z69881_at (ATP2A3), D38073_at (MCM3), U31556_at (E2F5), Z15115_at (TOP2B), U22376_cds2_s_at (MYB), L09209_s_at (APLP2), M12959_s_at (-), Y00787_s_at (IL8), M31211_s_at (MYL6B), U26266_s_at (DHPS), X85116_rna1_s_at (STOM), U49020_cds2_s_at (MEF2A), U72936_s_at (ATRX), J03801_f_at (LYZ), M19045_f_at (LYZ), M31523_at (TCF3), U27460_at (UGP2), U29175_at (SMARCA4)
* The significant genes were selected by *t*-test (*p*-value < 1.0e^-5^)

<Enriched GO terms>

| TermID | Term | Count | % | PValue |
| --- | --- | --- | --- | --- |
| GO:0002520 | immune system development | 8 | 12.5 | 1.88E-04 |
| GO:0042113 | B cell activation | 5 | 7.8125 | 3.31E-04 |
| GO:0030097 | hemopoiesis | 7 | 10.9375 | 5.50E-04 |
| GO:0045321 | leukocyte activation | 7 | 10.9375 | 6.28E-04 |
| GO:0048534 | hemopoietic or lymphoid organ development | 7 | 10.9375 | 9.14E-04 |
| GO:0042100 | B cell proliferation | 3 | 4.6875 | 1.41E-03 |
| GO:0006259 | DNA metabolic process | 9 | 14.0625 | 1.50E-03 |
| GO:0001775 | cell activation | 7 | 10.9375 | 1.52E-03 |
| GO:0002521 | leukocyte differentiation | 5 | 7.8125 | 2.53E-03 |
| GO:0002377 | immunoglobulin production | 3 | 4.6875 | 7.52E-03 |
| GO:0002440 | production of molecular mediator of immune response | 3 | 4.6875 | 8.02E-03 |

**Answer 2:**

KEGG04640: Hematopoietic cell lineage (pvalue = 1.9E-3)
KEGG05340: Primary immunodeficiency (pvalue = 1.9E -2)
KEGG04110: Cell cycle (pvalue = 1.9E-2)
KEGG04662: B cell receptor signaling pathway (pvalue = 1.9E-2)

**Answer 3:**

| KEGGID | Size | Global.Stat | Emp.pvalue |
| --- | --- | --- | --- |
| KEGG00860 | 15 | 110.39778 | 0.003 |
| KEGG04920 | 30 | 84.02642 | 0.011 |
| KEGG04110 | 51 | 99.95088 | 0.012 |
| KEGG00240 | 31 | 79.52313 | 0.022 |
| KEGG00564 | 10 | 66.96118 | 0.03 |
| KEGG00970 | 16 | 87.31489 | 0.03 |
| KEGG04640 | 70 | 94.99477 | 0.032 |

**Answer 4:**

R code:

> re1 <- ias(M, M1)
> re2 <- ias(M, M2)
> n1 <- n2 <- ncol(M)*(ncol(M)-1)/2
> re <- compcorr(n1, re1[[1]], n2, re2[[2]])

The difference of IS between the two conditions is13.56 and *p*-value of the difference is 0.

**Answer 5:**


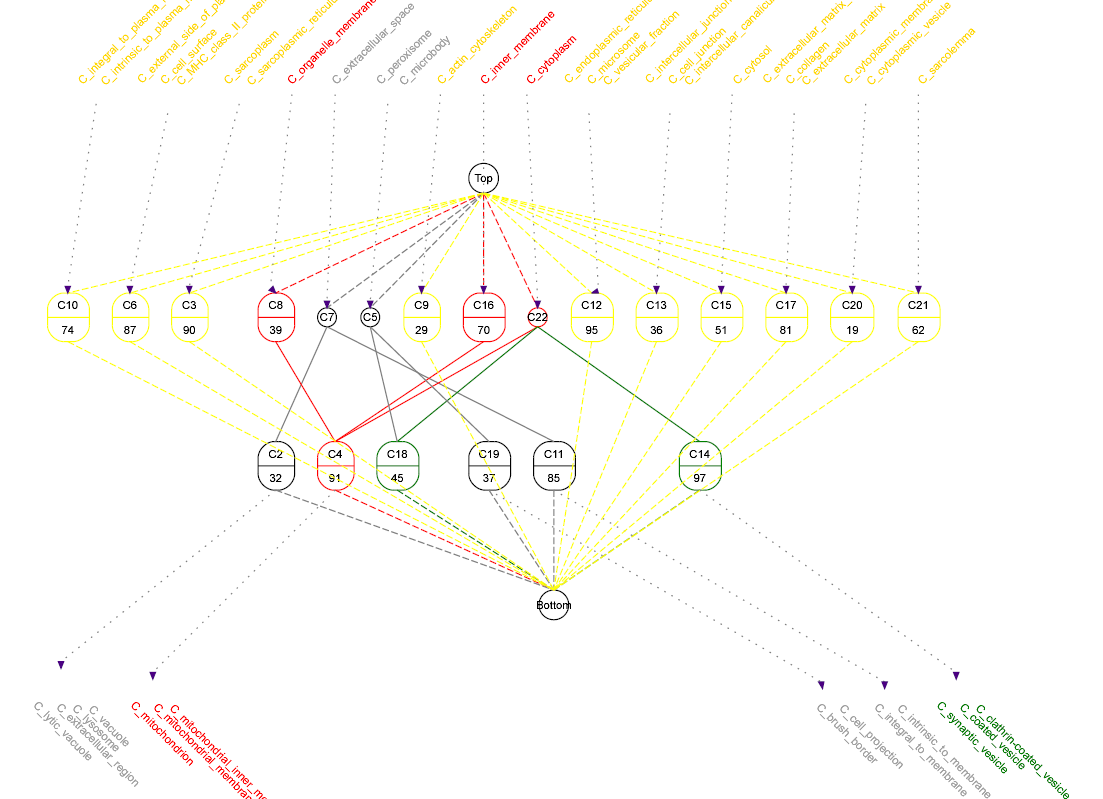


**Order Table for concepts from experiment**

| [**Concept 6**](http://cello.snubi.org/cgi-bin/biolattice/part.cgi?exp=test&concept=concept6) [**cluster 87**](http://cello.snubi.org/cgi-bin/biolattice/clsgene.cgi?cls=87&exp=test)  C_external_side_of_plasma_membrane C_cell_surface C_MHC_class_II_protein_complex | | |
| --- | --- | --- |
| [**Concept 21**](http://cello.snubi.org/cgi-bin/biolattice/part.cgi?exp=test&concept=concept21) [**cluster 62**](http://cello.snubi.org/cgi-bin/biolattice/clsgene.cgi?cls=62&exp=test)  C_sarcolemma | | |
| [**Concept 3**](http://cello.snubi.org/cgi-bin/biolattice/part.cgi?exp=test&concept=concept3) [**cluster 90**](http://cello.snubi.org/cgi-bin/biolattice/clsgene.cgi?cls=90&exp=test)  C_sarcoplasm C_sarcoplasmic_reticulum | | |
| [**Concept 7**](http://cello.snubi.org/cgi-bin/biolattice/part.cgi?exp=test&concept=concept7) [**cluster 32,85**](http://cello.snubi.org/cgi-bin/biolattice/clsgene.cgi?cls=32,85&exp=test)  C_extracellular_space | [**Concept 2**](http://cello.snubi.org/cgi-bin/biolattice/part.cgi?exp=test&concept=concept2) [**cluster 32**](http://cello.snubi.org/cgi-bin/biolattice/clsgene.cgi?cls=32&exp=test)  C_lytic_vacuole C_extracellular_region C_lysosome C_vacuole |  |
|  | [**Concept 11**](http://cello.snubi.org/cgi-bin/biolattice/part.cgi?exp=test&concept=concept11) [**cluster 85**](http://cello.snubi.org/cgi-bin/biolattice/clsgene.cgi?cls=85&exp=test)  C_integral_to_membrane C_intrinsic_to_membrane |  |
| [**Concept 9**](http://cello.snubi.org/cgi-bin/biolattice/part.cgi?exp=test&concept=concept9) [**cluster 29**](http://cello.snubi.org/cgi-bin/biolattice/clsgene.cgi?cls=29&exp=test)  C_actin_cytoskeleton | | |
| [**Concept 17**](http://cello.snubi.org/cgi-bin/biolattice/part.cgi?exp=test&concept=concept17) [**cluster 81**](http://cello.snubi.org/cgi-bin/biolattice/clsgene.cgi?cls=81&exp=test)  C_extracellular_matrix_(sensu_Metazoa) C_collagen C_extracellular_matrix | | |
| [**Concept 12**](http://cello.snubi.org/cgi-bin/biolattice/part.cgi?exp=test&concept=concept12) [**cluster 95**](http://cello.snubi.org/cgi-bin/biolattice/clsgene.cgi?cls=95&exp=test)  C_endoplasmic_reticulum C_microsome C_vesicular_fraction | | |
| [**Concept 20**](http://cello.snubi.org/cgi-bin/biolattice/part.cgi?exp=test&concept=concept20) [**cluster 19**](http://cello.snubi.org/cgi-bin/biolattice/clsgene.cgi?cls=19&exp=test)  C_cytoplasmic_membrane-bound_vesicle C_cytoplasmic_vesicle | | |
| [**Concept 15**](http://cello.snubi.org/cgi-bin/biolattice/part.cgi?exp=test&concept=concept15) [**cluster 51**](http://cello.snubi.org/cgi-bin/biolattice/clsgene.cgi?cls=51&exp=test)  C_cytosol | | |
| [**Concept 22**](http://cello.snubi.org/cgi-bin/biolattice/part.cgi?exp=test&concept=concept22) [**cluster 45,91,97**](http://cello.snubi.org/cgi-bin/biolattice/clsgene.cgi?cls=45,91,97&exp=test)  C_cytoplasm | [**Concept 4**](http://cello.snubi.org/cgi-bin/biolattice/part.cgi?exp=test&concept=concept4) [**cluster 91**](http://cello.snubi.org/cgi-bin/biolattice/clsgene.cgi?cls=91&exp=test)  C_mitochondrion C_mitochondrial_membrane C_mitochondrial_inner_membrane |  |
|  | [**Concept 14**](http://cello.snubi.org/cgi-bin/biolattice/part.cgi?exp=test&concept=concept14) [**cluster 97**](http://cello.snubi.org/cgi-bin/biolattice/clsgene.cgi?cls=97&exp=test)  C_synaptic_vesicle C_coated_vesicle C_clathrin-coated_vesicle |  |
|  | [**Concept 18**](http://cello.snubi.org/cgi-bin/biolattice/part.cgi?exp=test&concept=concept18) [**cluster 45**](http://cello.snubi.org/cgi-bin/biolattice/clsgene.cgi?cls=45&exp=test) |  |
| [**Concept 8**](http://cello.snubi.org/cgi-bin/biolattice/part.cgi?exp=test&concept=concept8) [**cluster 39,91**](http://cello.snubi.org/cgi-bin/biolattice/clsgene.cgi?cls=39,91&exp=test)  C_organelle_membrane | [**Concept 4**](http://cello.snubi.org/cgi-bin/biolattice/part.cgi?exp=test&concept=concept4) [**cluster 91**](http://cello.snubi.org/cgi-bin/biolattice/clsgene.cgi?cls=91&exp=test)  C_mitochondrion C_mitochondrial_membrane C_mitochondrial_inner_membrane |  |
| [**Concept 16**](http://cello.snubi.org/cgi-bin/biolattice/part.cgi?exp=test&concept=concept16) [**cluster 70,91**](http://cello.snubi.org/cgi-bin/biolattice/clsgene.cgi?cls=70,91&exp=test)  C_inner_membrane | [**Concept 4**](http://cello.snubi.org/cgi-bin/biolattice/part.cgi?exp=test&concept=concept4) [**cluster 91**](http://cello.snubi.org/cgi-bin/biolattice/clsgene.cgi?cls=91&exp=test)  C_mitochondrion C_mitochondrial_membrane C_mitochondrial_inner_membrane |  |
| [**Concept 10**](http://cello.snubi.org/cgi-bin/biolattice/part.cgi?exp=test&concept=concept10) [**cluster 74**](http://cello.snubi.org/cgi-bin/biolattice/clsgene.cgi?cls=74&exp=test)  C_integral_to_plasma_membrane C_intrinsic_to_plasma_membrane | | |
| [**Concept 13**](http://cello.snubi.org/cgi-bin/biolattice/part.cgi?exp=test&concept=concept13) [**cluster 36**](http://cello.snubi.org/cgi-bin/biolattice/clsgene.cgi?cls=36&exp=test)  C_intercellular_junction C_cell_junction C_intercellular_canaliculus | | |
| [**Concept 5**](http://cello.snubi.org/cgi-bin/biolattice/part.cgi?exp=test&concept=concept5) [**cluster 37,45**](http://cello.snubi.org/cgi-bin/biolattice/clsgene.cgi?cls=37,45&exp=test)  C_peroxisome C_microbody | [**Concept 18**](http://cello.snubi.org/cgi-bin/biolattice/part.cgi?exp=test&concept=concept18) [**cluster 45**](http://cello.snubi.org/cgi-bin/biolattice/clsgene.cgi?cls=45&exp=test) |  |
|  | [**Concept 19**](http://cello.snubi.org/cgi-bin/biolattice/part.cgi?exp=test&concept=concept19) [**cluster 37**](http://cello.snubi.org/cgi-bin/biolattice/clsgene.cgi?cls=37&exp=test)  C_brush_border C_cell_projection |  |
